# Automated detection of hippocampal sclerosis using real-world clinical MRI images

**DOI:** 10.3389/fnins.2023.1180679

**Published:** 2023-05-15

**Authors:** Jingwen Jiang, Jiajun Qiu, Jin Yin, Junren Wang, Xinyue Jiang, Zuo Yi, Yang Chen, Xiaobo Zhou, Xiutian Sima

**Affiliations:** ^1^Department of Neurosurgery and West China Biomedical Big Data Center, West China Hospital of Sichuan University, Chengdu, China; ^2^Med-X Center for Informatics, Sichuan University, Chengdu, China; ^3^Department of Radiology, Chengdu Second People's Hospital, Chengdu, China; ^4^Department of Computer Science and Technology, College of Computer Science, Sichuan University, Chengdu, China; ^5^School of Biomedical Informatics, University of Texas Health Science Center at Houston, Houston, TX, United States; ^6^Department of Neurosurgery, West China Hospital of Sichuan University, Chengdu, China

**Keywords:** real-world clinical MRI images, hippocampal sclerosis, fractional differential, deep learning, computer aided diagnosis

## Abstract

**Background:**

Hippocampal sclerosis (HS) is the most common pathological type of temporal lobe epilepsy (TLE) and one of the important surgical markers. Currently, HS is mainly diagnosed manually by radiologists based on visual inspection of MRI, which greatly relies on MRI quality and physician experience. In clinical practice, non-thin MRI scans are often used due to the time and efficiency needed for the acquisition. However, these scans can be difficult for junior physicians to interpret accurately. Thus, the rapid and accurate diagnosis of HS using real-world MRI images in clinical settings is a challenging task.

**Objective:**

Our aim was to explore the feasibility of using computer vision methods to diagnose HS on real-world clinical MRI images and to provide a reference for future clinical applications of artificial intelligence methods to aid in detecting HS.

**Methods:**

We proposed a deep learning algorithm called “HS-Net” to discriminate HS using real-world clinical MRI images. First, we delineated and segmented a region of interest (ROI) around the hippocampus. Then, we utilized the fractional differential (FD) method to enhance the textures of the ROIs. Finally, we used a small-sample image classification method based on transfer learning to fine-tune the feature extraction part of a pretrained model and added two fully connected layers and an output layer. In the study, 96 TLE patients with HS confirmed by postoperative pathology and 89 healthy controls were retrospectively enrolled. All subjects were cross-validated, and models were evaluated for performance, robustness, and clinical utility.

**Results:**

The HS-Net model achieved an area under the curve (AUC) of 0.894, an accuracy of 82.88%, an F1-score of 84.08% in the test cohort based on real, routine, clinical T2-weighted fluid attenuated inversion recovery (FLAIR) sequence MRI images. Additionally, the AUC, accuracy and F1 scores of our model all increased by around 3 percentage points when the inputs were augmented with the ROIs of the textures enhanced using the FD method.

**Conclusions:**

Our computational model has the potential to be used for the diagnosis of HS in real clinical MRI images, which could assist physicians, particularly junior physicians, in improving the accuracy of discrimination.

## 1. Introduction

Epilepsy is one of the most common chronic neurological diseases, affecting more than 70 million people worldwide, accounting for 0.5% of the global disease burden, and affecting a broad population of people of all ages, races, social classes and geographic locations (Fiest et al., 2017; Feigin et al., 2019; Thijs et al., 2019; Trinka et al., 2019; Beghi, 2020). For most patients with epilepsy, treatment with antiepileptic drugs is the mainstay of treatment, with the aim of stopping seizures as early as possible without causing side effects that can affect quality of life. However, more than half of patients taking epilepsy drugs still have seizures, according to surveys in the United States in 2013 and 2015 (Tian et al., 2018). Although antiepileptic drugs may suppress seizures in up to two-thirds of patients, up to one-third of patients with epilepsy may still have drug-resistant epilepsy. For drug-resistant epilepsy, especially for focal epilepsy, surgical resection of the epileptogenic foci may be a more effective method. With surgery to remove or disconnect restrictive brain regions, patients can achieve complete seizure control or at least stop them. In carefully selected groups, 50–80% of individuals were seizure-free after surgery (Ryvlin et al., 2014). Surgery appears to be cost-effective and superior to optimal medical therapy in terms of epilepsy control and quality of life (Wiebe et al., 2001; Engel et al., 2012; Picot et al., 2016; Dwivedi et al., 2017). The benefits of successful surgery also include a reduced risk of injury or premature death, opportunities to drive, greater independence, and potentially improved career choices. Therefore, surgical treatment decisions are critical for the treatment of drug-resistant focal epilepsy.

Temporal lobe epilepsy (TLE) in drug-resistant focal epilepsy is the most common type of epilepsy in children and adults (Goubran et al., 2016). TLE is mostly associated with lesions of the temporal cortex, and the most common pathological type is hippocampal sclerosis (HS), accounting for approximately 50–83% of TLE cases (Mueller et al., 2007). More than 70% of HS epilepsy patients can be cured by surgical resection of the hippocampus (Granados Sanchez and Orejuela Zapata, 2018). Therefore, HS serves as a major histopathological hallmark and major underlying etiology of TLE (Blumcke et al., 2017). Notably, misdiagnosis of HS early in the disease course may lead to surgical delays, which are associated with cumulative brain damage, cognitive decline, and increased risk of disability and death, as well as significant socioeconomic consequences (Wiebe et al., 2001). Therefore, one of the keys to choosing a surgical treatment path for TLE is to quickly and accurately discriminate HS.

At present, MRI is mainly used as a standard imaging tool to detect and diagnose epilepsy foci, and more than half of patients with drug-resistant focal epilepsy can be diagnosed with epileptogenic foci (Berg et al., 2009; Hakami et al., 2013; Duncan et al., 2016). Among them, the imaging features of HS on MRI may include marked atrophy on coronal T1-weighted images, hyperintensity on T2-weighted and FLAIR images, and loss of definition of the internal structures of the hippocampus (Coras et al., 2014). In the diagnosis of HS in China, radiologists mainly use MRI to visually diagnose HS and perform a visual inspection or quantitative measurement of lesions such as hippocampal atrophy and hippocampal signal increase. The accuracy of diagnosis depends on the doctor's experience and imaging quality. Physicians with imaging experience in diagnosing epilepsy are quite different in terms of diagnosing HS from those with little or no relevant experience (Azab et al., 2015). Regarding the quality of MRI, some studies have shown that the performance of 3.0T MR in detecting HS is better than that of 1.5T MR (Coan et al., 2014).

Considering that in the actual diagnosis process, especially in primary hospitals, clinical facilities rarely have 3.0T and higher-performance MR instruments, the conventional acquisition equipment is 1.5T MR, and most of the obtained medical images are of low resolution (LR). In the initial screening test, considering acquisition time, cost, and efficiency, conventional MR imaging sequences have mainly been used, with slice thicknesses ranging from 3 to 10 mm, with intervals, and few thin-slice sequences (i.e., slice thicknesses ≤ 1 mm) without intervals. Furthermore, as for the doctors' experience, it is impossible for primary hospital physicians or junior physicians to have enough solid experience to accurately discriminate HS. This is a great challenge for physicians in primary hospitals or junior doctors to diagnose HS with conventional MR sequences, while it is the key to whether patients can be promptly transferred to high-level hospitals or undergo surgical treatment.

With the development of computer vision technology and artificial intelligence, there are an increasing number of studies using computer-aided discrimination of HS. Current studies mainly use MRI sequences with thin thickness ( ≤ 1 mm thickness) of good quality 3T MR images to extract imaging histology features and later construct machine learning classification models (Mo et al., 2019). Other studies have used computer vision techniques to automatically measure features such as hippocampal volume and symmetry in MRI and construct machine learning classification models to discriminate HS (Mettenburg et al., 2019). Furthermore, some studies have used deep learning to reconstruct low-resolution MRI images into high-resolution images for HS differential diagnosis (Cao et al., 2021). Based on our knowledge, no studies based on real clinical MRI common sequences using computer vision or deep learning to discriminate HS have been published.

This study attempted to mimic the real clinical diagnosis process of HS, construct a deep learning model, namely, HS-Net, using real-world clinical routine MRI sequences with pathological findings as the gold standard to assist primary hospital physicians or junior doctors in rapidly discriminating HS in patients with TLE, explore the feasibility of using deep learning algorithms to discriminate HS from conventional MRI sequences, and provide radiological evidence for the actual clinical identification of HS.

## 2. Materials and methods

### 2.1. Study design

**Figure 1:**
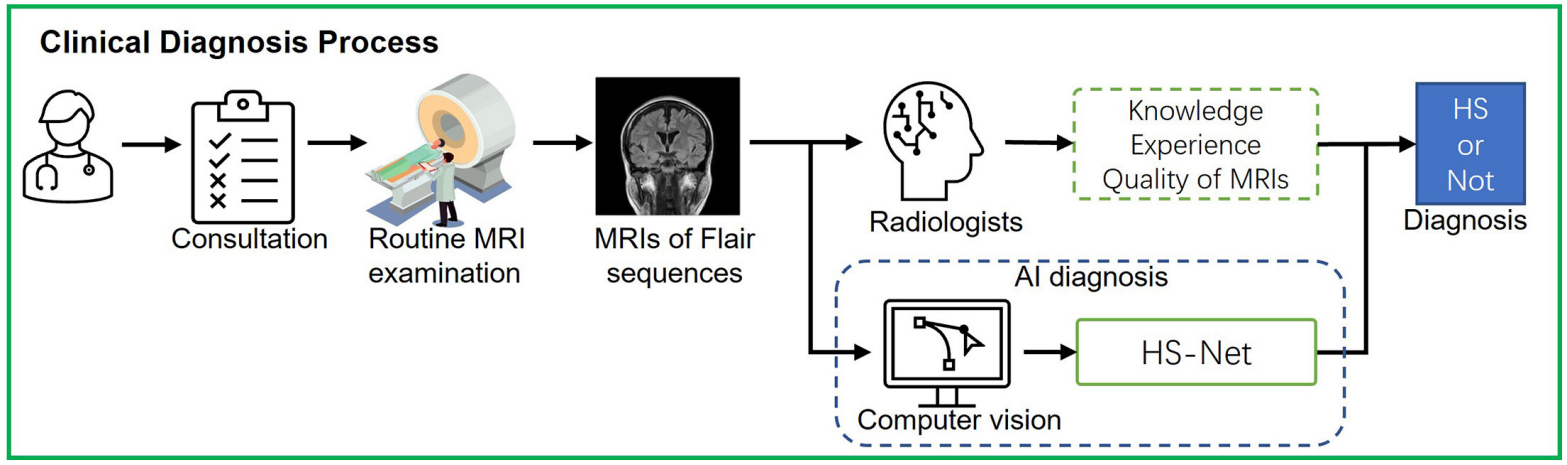
The process of real clinical diagnosis and AI-assisted diagnosis for HS discrimination.

This study explores the use of computer vision to assist clinicians in discriminating HS based on a real clinical diagnostic process. As shown in Figure 1, in the actual diagnostic process, the physician first asks the patient about his or her condition and determines whether a head MRI is needed. If there are no special symptoms or needs, the doctor will prescribe a non-thin, routine head MRI in consideration of acquisition time and efficiency in most cases. Subsequently, the radiologist will determine whether the patient has HS by observing MRI findings based on his or her knowledge and experience. The accuracy of HS diagnosis relies on MRI quality and physician experience and is prone to miss milder lesions in bilateral HS, mild HS, and focal abnormalities of the hippocampus. Junior doctors may not have enough good experience to discriminate HS. We therefore designed an artificial intelligence (AI)-assisted diagnostic module in the HS discrimination stage and built a computer vision-based deep learning network namely HS-Net to assist radiologists (especially junior radiologists) in the discrimination of HS. This study design was close to clinical practice, using clinically real MRIs for HS discrimination, rather than thin-layer high-resolution MRIs collected intentionally for research.

### 2.2. Data source and study population

The Ethics Committee of the West China Hospital, Sichuan University approved the research. The Institutional Review Board (IRB) did not require informed consent from the patients. Because this was a retrospective study, we did not use any identifying information of the patients. Moreover, we kept the patient information confidential. The protection and treatment of patient data in our research complied with the Helsinki Declaration.

We used the database from Hospital Information System (HIS) and Picture Archiving and Communication System (PACS) from West China Hospital of Sichuan University. The database includes inpatient and outpatient diagnoses and MRI images. Referring to the inclusion criteria of other studies (Mo et al., 2019), participants were retrospectively selected from the dataset of patients with drug-resistant mesial TLE (mTLE) between 2009 and 2020 according to the following inclusion criteria:

1. Anterior temporal lobectomy or selective amygdalohippocampectomy;
2. Resected hippocampal specimen suitable for histological analysis based on the International League Against Epilepsy HS classification scheme (Blümcke et al., 2016);
3. A definite postoperative histopathological diagnosis of hippocampal sclerosis;
4. Presurgical general MRI scans including at least sagittal or coronal T2-FLAIR images without motion artifacts, aliasing, or rippling related to eye movement.

At the same time, we excluded patients with any of the following conditions:

1. Type III focal cortical dysplasia (FCD) on histopathology (e.g., HS with FCD in the temporal lobe);
2. History of dystocia hypoxia, encephalitis, or severe traumatic brain injury;
3. Intracranial lesions (malformations of cortical development, epidermoid cysts, tumors, vascular malformations);
4. Encephalomalacia and no severe or diffuse brain atrophy;
5. Reoperations.

For the control group, healthy normal controls (HCs) with no history of any neurological disorders and no MRI abnormalities were selected. All participants had the following clinical information in the current study: age, sex, and lateralization of the affected hippocampus.

### 2.3. MRI acquisition and ROIs enhancing

MRIs in all participants were acquired on a 1.5-T Siemens Verio scanner including a T2-FLAIR sequence. Because the slice thicknesses of plain sequences were between 3 and 7 mm, the number of MRI slices containing hippocampal regions varied for each subject. Because this study focused on the hippocampus, we selected only MRI slices that contained the hippocampal region and depicted a region of interest (ROI) along the edge of the hippocampus. For HS patients, we depicted only the hippocampus with HS, while for HCs, we randomly depicted one hippocampus. To better match the actual clinical diagnostic process, slice selection and ROI mapping were performed by two junior doctors (< 3 years). To reduce measurement bias and ensure accuracy, a surgeon with 10 years of experience in epileptic HS surgery examined the slice selection and ROI depiction. Such slice selection and ROI depiction mimicked the perspective of the junior doctors at the time of diagnosis, allowing the ROI in the deep learning model to be consistent with the ROI observed by the junior surgeon. We counted the number of slices included per subject and the lateralization of the affected hippocampus in HS patients. To facilitate the training of the subsequent models, we used a rectangular box to segment the ROI region and resize the rectangular image to 224*224 size as the input of the deep learning network.

We designed a texture enhancement method with reference to the Grunwald–Letnikov (G-L) fractional differential (FD) definition (De Oliveira and Tenreiro Machado, 2014) to enhance the textures of the rectangular ROIs. The enhancement process involves constructing a fractional differential operator and convolving each ROI with this operator. First, we construct the fractional differential operator using three equations as:


(1) $${}_{a}Dt^{v}f(x)=\underset{h\to 0}{lim}h^{-v}\sum_{j=0}^{(t-a)/h}{\left(-1\right)}^{j}\frac{\Gamma (v+1)}{j!\Gamma (v-j+1)}f(x-jh)$$

(2) $${}_{a}Dt^{v}f(x)=\frac{1}{h^{v}}\sum_{j=0}^{n-1}{\left(-1\right)}^{j}\frac{\Gamma (j-v)}{\Gamma (-v)\Gamma (j+1)}f(x-jh)$$

(3) $$\begin{array}{l} \begin{array}{l} \frac{d^{v}f(x)}{dx}\approx f(x)+(-v)f(x-1)+\frac{\left(-v)(-v+1\right)}{2}f(x-2) \end{array}\\ \;\begin{array}{l} +\cdots +\frac{\Gamma (-v+1)}{\left(n-1)!\Gamma (-v+n)f(x-n+1\right)} \end{array} \end{array}$$

**Figure 2:**
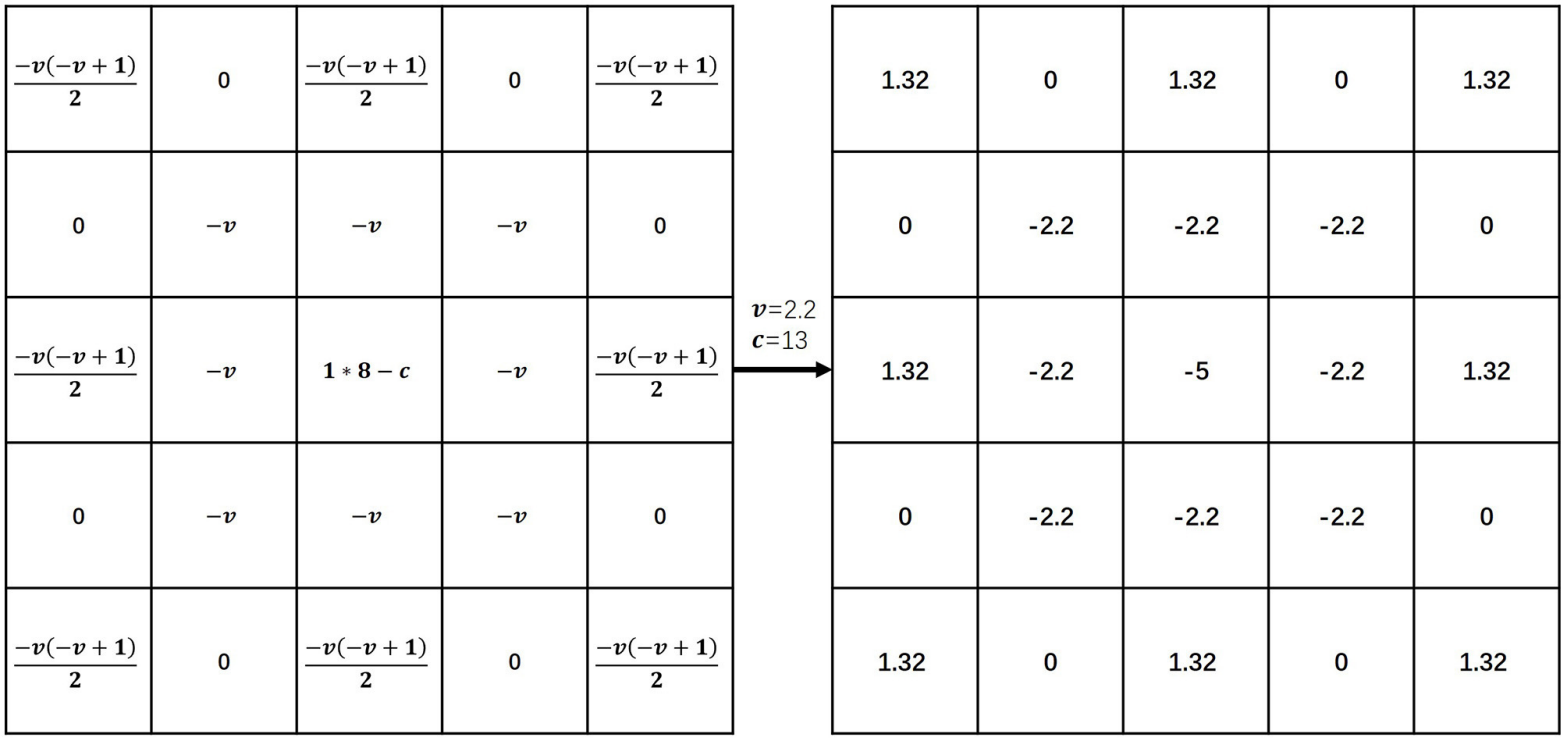
Fractional differential operator. Illustration inspired by Hui et al. (2020). In our experiments, the order *v* was set to 2.2 and the parameter *c* was set to 13. The coefficient of *f*(*x*) was “1”, the coefficient of *f*(*x*−1) was *v* and the coefficient of *f*(*x*−2) was $\frac{\left(-v\right)\left(-v+1\right)}{2}$.

Equation (1) represents the *v*-order G-L definition of *f*(*x*) on [*a, t*], where Γ(·) is a gamma function. Equation (2) is the discretized form of the G-L definition which divides the continuous interval [*a, t*] equally into unit intervals h, where *n* = (*t*−*a*)/*h*. Equation (3) is the expansion of Equation (2) where *h* = 1 (unit interval) is known. We constructed the fractional differential operator based on the expanded coefficients of Equation (3), following the construction of the fractional differential mask (Hui et al., 2020). We show a fractional differential operation in eight symmetric directions of a 5 × 5 neighborhood in Figure 2. The parameter *c* at the center point position is referred to as the compensation parameter. The two parameters *v* and *c* are adjustable. In our experiments, the order *v* was set to 2.2 and the parameter *c* was set to 13.

**Figure 3:**
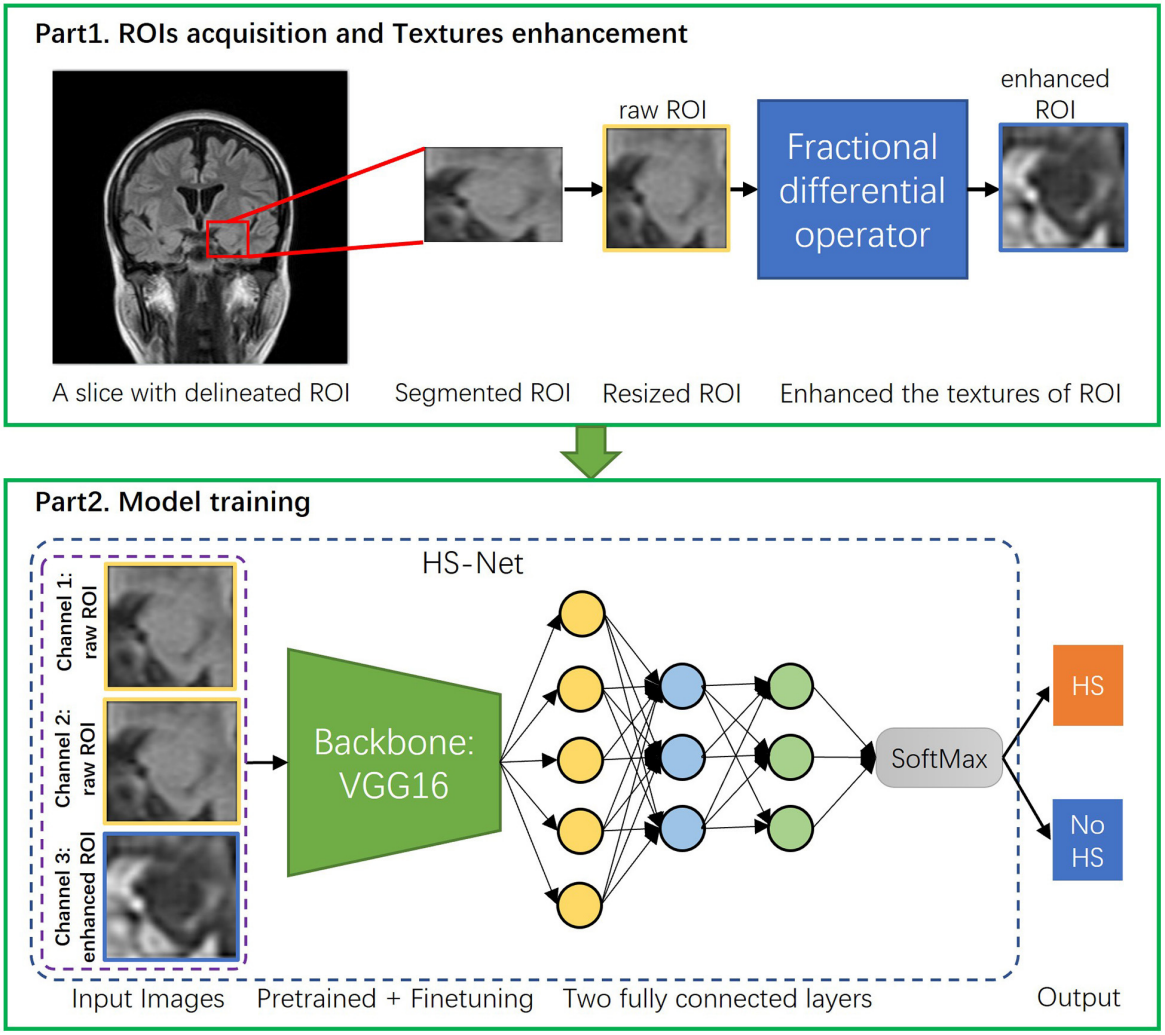
The overall procedure of the proposed pipeline. The proposed pipeline consists of two parts: (1) Acquisition of ROIs of hippocampal regions and enhancement of the ROIs' textures using the fractional differential (FD) method. (2) Construction of the HS-Net model, including the use of input images with two channels using raw ROIs and one channel using enhanced ROIs, a backbone network for fine-tuning feature extraction, and two fully connected layers plus a SoftMax activation function to discriminate HS.

To summarize, Figure 3 part 1 illustrates the entire image preprocessing process of the HS-Net model.

### 2.4. Model structure, training process, and implementation

To discriminate hippocampal sclerosis, our HS-Net model uses a classical convolutional neural network, VGG16 (Simonyan and Zisserman, 2014), as the backbone network, which is commonly used in deep learning frameworks for image classification. By using multiple small convolutional kernels, VGG16 automatically mines the deep features of an image while expanding the receptive field. After all convolutional layers of the VGG16 network, we put the extracted deep features of the hippocampal region into 2 fully connected layers and a SoftMax activation function to discriminate HS. In Figure 3 part 2, we show the model structure and training process. At the same time, we also use two other lightweight classical CNN frameworks, ResNet18 (He et al., 2016) and MobileNetV2 (Sandler et al., 2018) as comparisons to select the best results.

In this study, our model training process contained two strategies that contributed to the accuracy of the results: loading pretrained models and fine-turning.

Due to the sample size limitation, it was difficult for us to train all parameters of the deep learning network from scratch. To make our model have a certain image recognition ability before training, we chose the pretrained VGG16 model with weight parameters from the large image dataset ImageNet (Deng et al., 2009). Many research experiments show that by using the low and middle layers of the pretrained model as feature extractors and the top layer or near top layer of the model as classifiers, the image classification accuracy can be improved to some extent.

Additionally, a fine-tuning strategy was used in this study. We froze all the convolutional layers in the early training phase when the learning rate was high and only fine-tuned the final fully connected layer. As the learning rate decreased to a certain level and the loss function became more stable, we allowed the whole network to undergo some fine-tuning by no longer freezing the convolutional layers.

In the actual training of the model, we treated each ROI slices as a separate sample for model training. We used all ROI slices as the total data set, with 80% of the ROI slices as the training set and the remaining 20% of the ROI slices as the test set. In particular, the ROI slices of the same subject were either all classified as training data or all classified as test data, avoiding the model accuracy overestimation caused by the ROI slices of the same subject being partly used for training and partly used for testing. In the training set, we performed 10-fold cross-validation to validate the results. Then we tested the results in the testing set which was not at all involved in the training. To address the issue of insufficient training samples and improve the model's generalization ability, this study implemented a random preprocessing of data augmentation (Shorten and Khoshgoftaar, 2019) by horizontal flipping or scaling the training ROI slices. Besides, raw ROI slices are grayscale maps with only one channel. However, for VGG16, ResNet18, and MobileNetV2 models, the input images should have three channels. To meet this requirement, we copy each original ROI slice once as the second channel of the input, and the corresponding ROI slice enhanced by FD as the third channel. As a result, we construct a three-channel input image for each sample that includes the two original ROI slices and the corresponding ROI slice enhanced by FD.

The model was trained using the Adam algorithm to optimize the loss function of the updated network parameters. The training process used 80 epochs in total. In the first 40 epochs, the convolutional layers were frozen, and the batch size was set to 32, while in the last 40 epochs the convolutional layers were unfrozen, and the batch size was set to 24. The learning rate (LR) was initially set to 0.00001 and used an exponential decay strategy. All experiments were completed in 2 h on one Tesla V100 GPU. All codes were implemented based on the PyTorch framework. The overall procedure of the proposed pipeline in this study is shown in Figure 3.

## 3. Results

### 3.1. Basic information

**Figure 4:**
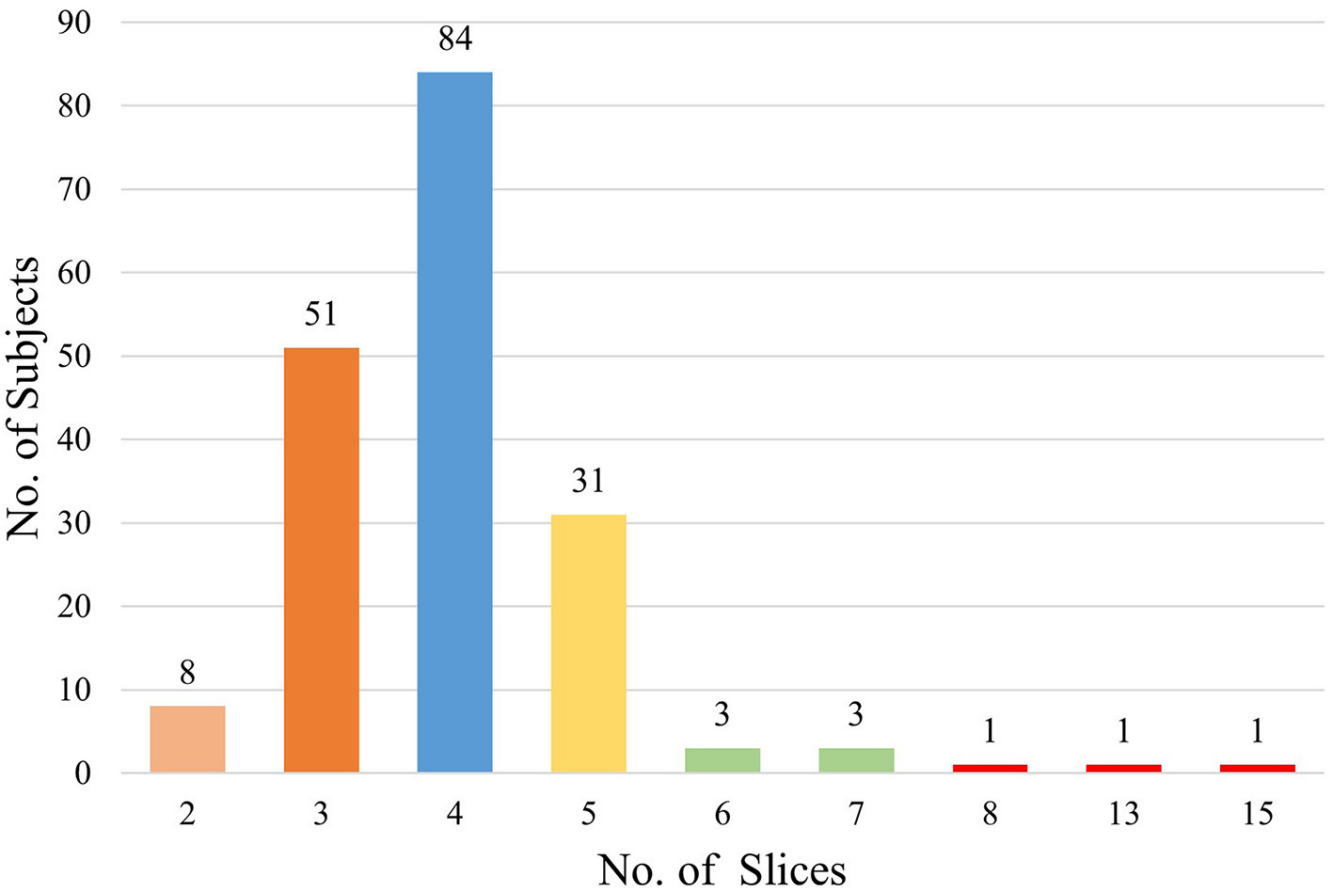
Distribution graph of the number of slices.

A total of 183 subjects were included in this study according to the inclusion and exclusion criteria, and a total of 735 MRI slices containing hippocampal regions were screened. Among the subjects, there were 94 patients with HS, including 396 slices, and 89 HCs, including 339 slices. The basic information of these subjects is shown in Table 1. The number of MRI slices containing hippocampal regions for each subject ranged from 2 to 15, and the distribution of the number of slices is shown in Figure 4.

**Table 1:** Basic characteristics of the HS patients and HCs.

	**HS**	**HC**	**Total**
No. of subjects	94	89	183
**Sex**			
Female	49	50	99
Male	45	39	84
Age [Mean (SD)]	26.49 (9.00)	47 (17.27)	36.47 (17.08)
No. of slices	396	339	735
**Lateralization of the**			
**affected hippocampus**			
Left	204	178	382
Right	192	161	353

SD, standard deviation.

### 3.2. Model results

In this section, we presented the results of the proposed method applied on the test set including a comparison study of different backbone networks and an ablation study of enhancing ROIs through fractional differential. To facilitate the subsequent description, we denoted the proposed HS-Net as “HS-Net(CNN+FD)”, which represents an HS-Net that uses a CNN backbone network with enhanced ROIs using the FD method. We also referred to “HS-Net(CNN)” as an HS-Net that uses a CNN backbone network and does not include the enhanced ROIs.

Firstly, we trained the HS-Net model using VGG16 as the backbone network to discriminate HS. We also used ResNet18 and MobileNetV2 as the backbone networks for HS-Net models to compare the results. Table 2 showed the results for the HS-Net models using the three backbone networks. Table 2 shows the results for each of these models. The HS-Net model using VGG16 with enhanced ROIs [HS-Net(VGG16+FD)] achieved the best performance with 82.88% accuracy, 84.08% F1 score, and 0.894 AUC. The HS-Net model using ResNet18 with enhanced ROIs [HS-Net(ResNet18+FD)] achieved the second-best performance, with an AUC and accuracy that were lower than HS-Net(VGG16+FD) by 5 and 2 percentage points, respectively. The HS-Net model using MobileNetV2 with enhanced ROIs [HS-Net(MobileNetV2+FD)] had the worst performance.

**Table 2:** The results of the HS-Net models in discriminating HS.

	**AUC**	**Accuracy (%)**	**F1 core (%)**	**Precision (%)**	**Recall (%)**
**With enchanced ROIs**					
HS-Net (VGG16+FD)	**0.894**	**82.88**	**84.08**	84.62	83.54
HS-Net (ResNet18+FD)	0.842	80.82	80.82	**88.06**	74.68
HS-Net (MobileNetV2+FD)	0.827	79.45	81.93	78.16	**86.08**
**Without enchanced ROIs**					
HS-Net (VGG16)	0.859	80.14	81.53	82.05	81.01
HS-Net (ResNet18)	0.836	77.40	78.71	80.26	77.22
HS-Net (MobileNetV2)	0.778	74.66	74.48	81.82	68.35

FD, fractional differential. The bold values in the table represent the optimal results within each column.

**Figure 5:**
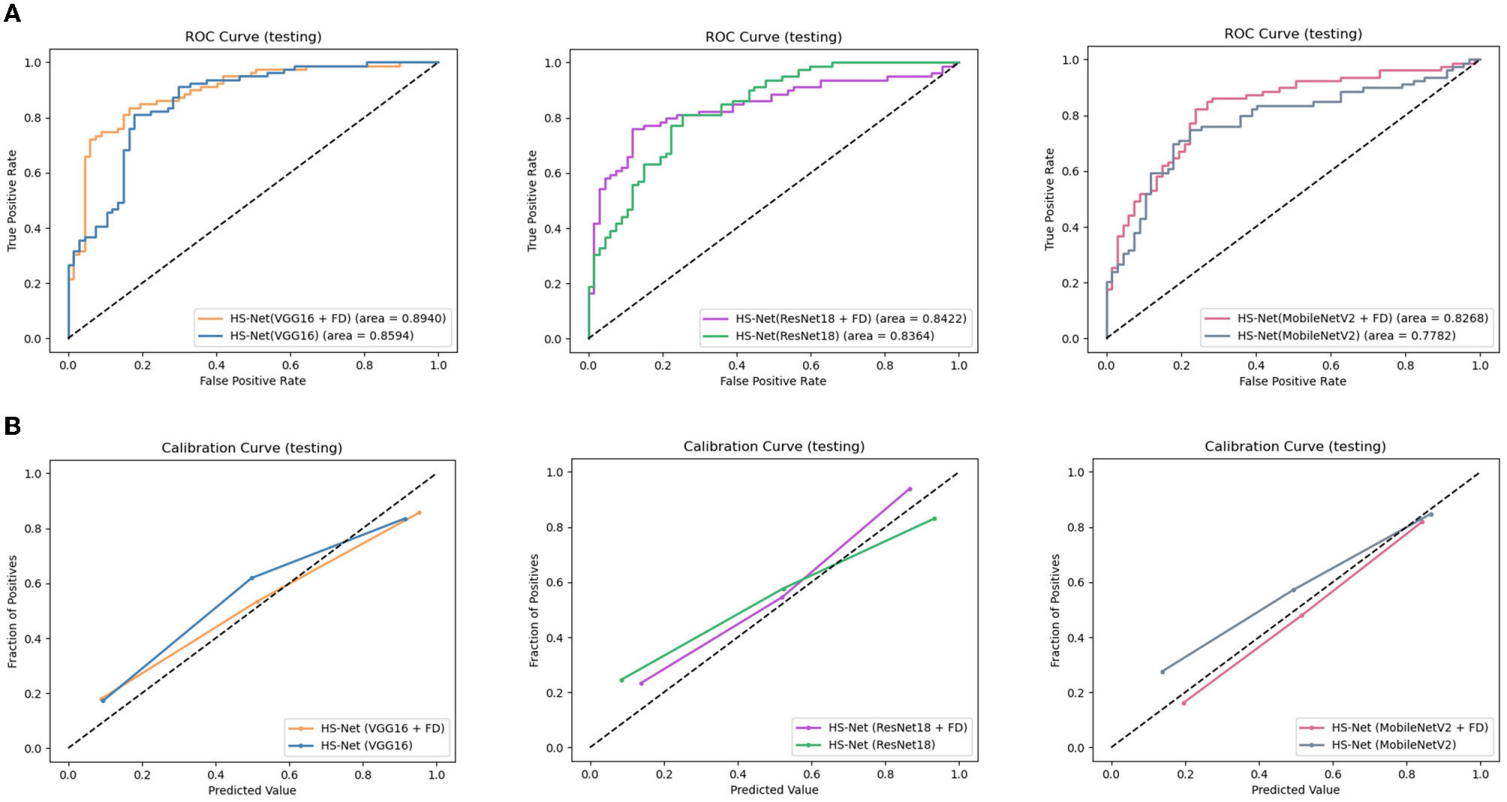
Evaluation of the proposed models: **(A)**The receiver operating characteristic (ROC) curves of the HS-Net models, with a larger area under the curve (AUC) indicating better model classification prediction. **(B)** The calibration curves of the HS-Net models, with a curve closer to the diagonal indicating more accurate model classification predictions. FD, fractional differential.

Additionally, we conducted an ablation study to evaluate the impact of enhancing the texture of ROIs by the FD method on the performance of the HS-Net model. The results in Table 2 shows that the accuracy and F1 score of all three models decreased when the inputs removed the enhanced ROIs. Specifically, the accuracy and F1 score of the HS-Net model using Vgg16 or ResNet18 decreased by almost 3 percentage points, while using MobileNetV2 decreased by almost 5 percentage points. Figure 5A illustrates the ROC curves for comparing the HS-Net models with and without enhanced ROIs. All three models with enhanced ROIs achieved better AUCs than the models without enhanced ROIs, with only the difference between the ROC curve of HS-Net(ResNet18+FD) and HS-Net(ResNet18) being less than one percentage point. This suggests that the fractional differential (FD) method is an effective technique for enhancing the texture of ROIs and improving the performance of the HS-Net model. Figure 5B shows the calibration curves to compare the performance of the HS-Net model using three different backbone networks and the effect of inputs with and without enhanced ROIs on the model performance in the HS-Net under each backbone network. The calibration curves of the HS-Net models were all close to the diagonal, and the HS-Net models with enhanced ROIs were closer to the diagonal. In the Supplementary material, we present a comparison of the effects of different values of *v* and *c* in the FD method on the experimental results in Supplementary Table 1.

**Figure 6:**
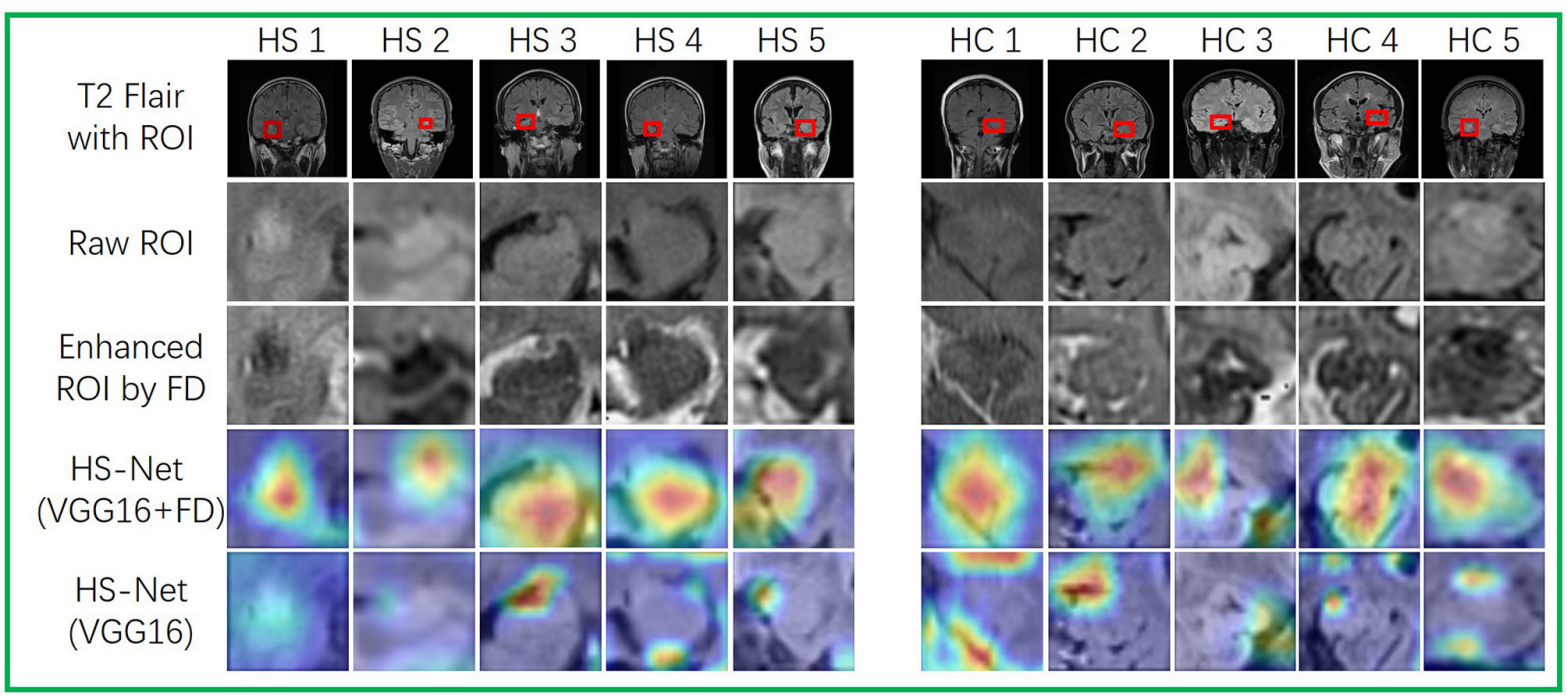
Visualizations of ROIs and attention maps for several test images. The first three rows depict raw MRIs from T2 flair sequences with ROIs. The first row displays the raw MRIs with ROIs, while the second row shows the resized raw ROIs. The third row depicts the enhanced ROIs, which have been processed using the FD method. The last two rows present attention maps that use Grad-CAM to highlight the different attention areas between HS-Net(VGG16) and HS-Net(VGG16+FD). A deep red denotes high attention.

To investigate the influence of enhanced ROIs using the FD method on attention maps for HS-Net, we utilized the gradient-weighed class activation mapping (Grad-CAM) method described in Selvaraju et al. (2017), Aggarwal et al. (2023) to visualize the attention maps of HS-Net(VGG16+FD) and HS-Net(VGG16). To generate the attention maps, we first derived the Grad-CAMs of the last convolution layer before the fully connected layers in HS-Net and then projected these weighed Grad-CAMs back to the raw ROIs based on their original coordinates. Additionally, we displayed the raw ROIs from the T2 flair sequence MRI and the enhanced ROIs using the FD method, providing a clear comparison between the two. Figure 6 shows these ROIs and attention maps at the same time. As shown in the first three rows of Figure 6, the ROIs enhanced using the FD method exhibited an inverted gray level that complemented the gray features of the raw ROIs. Moreover, the FD method enhanced the details of the gray areas in the raw ROIs, particularly when the gray areas were dominant and lacked distinctive features, such as texture features. As shown in the last two rows of Figure 6, the attention areas of HS-Net(VGG16+FD) are more concentrated and larger than those of HS-Net(VGG16). Furthermore, the attention areas of HS-Net(VGG16) are more affected by the gray level of the original image, with a greater focus on the junctions where gray changes are more evident, while disregarding the white matter area where the gray changes are less obvious.

Overall, our proposed HS-Net model using the VGG16 framework as the backbone network and enhanced ROIs by the fractional differential method showed promising results in the discrimination of HS in clinical routine MRI images.

## 4. Discussion

In this retrospective study, we found that a deep learning algorithm based on real clinical MRI common sequences performed moderately well in discriminating HS. The algorithm in the test had an AUC of 0.89 and an accuracy of 82.88%. In addition, The HS-Net models using the other two classical CNN backbone network in this study, ResNet18 and MobileNetV2, had a slightly lower performance than the HS-Net model using VGG16, but both achieved moderate performance for the feasibility goal of this study. These results suggest that it is feasible to use deep learning algorithms on real clinical MRI common sequences to assist in the discrimination of HS.

The main purpose of texture enhancement is to highlight detailed information and is a very important data augmentation technique for MRI images with poor quality in real clinical situations. Traditional enhancement methods, such as histogram equalization, integer-order differential techniques, and frequency enhancement filters, increase contrast or highlight contours, but they often result in the loss of significant low frequency texture information and tend to sharpen contour information (Hui et al., 2020). In contrast, fractional differentials have been shown to effectively compensate for this drawback by preserving low-frequency information, making them an effective method for enhancing texture of medical images (Jalab and Ibrahim, 2013; Li and Xie, 2015; Wang et al., 2019). Thus, we consider using fractional differential to enhance our ROIs. From our experimental results, it can be seen that the use of ROIs enhanced by the FD method indeed improved the performance of the model, regardless of which feature extraction network was used in the model. Our ROIs specifically target the hippocampal region, which is inherently small in volume and hence, can provide limited information to the model. However, as depicted in Figure 6, the ROIs enhanced by the FD method contain significantly more texture details compared to the raw ROIs. Combining the FD method-enhanced ROIs with the raw ROIs for model training equates to providing the model with better quality images and more detailed information, which ultimately improves the accuracy of the model. Furthermore, it is clear from Figure 6 that the model with FD-enhanced ROIs has a higher concentration of highlighted regions in the attention map, focusing not only on the transition regions with significant grayscale changes but also on the regions where the texture is enhanced by FD. It can be seen that the addition of FD-enhanced ROIs can indeed help the model to learn the features of the hippocampal sclerotic region.

The number of MRI slices containing hippocampal regions of each subject in the study is shown in Figure 4. Most of the MRI slices of the subjects had only 3–5 slices containing hippocampal regions in real clinical MRI sequences. Among them, the minimum number of slices containing hippocampal regions was only 2 per person, while the maximum number of slices containing hippocampal regions was 15 per person. It seems that in the actual clinical diagnosis of HS, physicians do not require patients to undergo MRI thin sequence scans, and there are no standards to specify the MRI slice thickness and interval width used for HS diagnosis. However, existing discriminatory studies of HS have largely used scientifically finely designed high-resolution MRI thin-layer sequences. For example, Mo et al. (2019) reported the use of radiomics and machine learning algorithms to discriminate HS on high-resolution MRI sequences with a layer thickness of 1 mm and no interval scans with an AUC of more than 99%. Although the results of Mo et al. (2019) are better than those of our investigation, such studies require fine experimental material that cannot be easily used in the clinical setting. This limits the generality and generalizability of HS discriminant studies. Our study can compensate for the lack of practical clinical application since we used real clinical MRIs and is well-suited to be developed into a clinical tool that can be flexibly embedded into a diagnostic system to assist junior doctors or primary hospital physicians in real-world clinical diagnosis of HS.

Since our main goal of this study is to explore the feasibility of deep learning algorithms to discriminate HS based on real clinical MRI common sequences, the studies all use a very classical lightweight CNN structure. With the development of deep learning algorithms, we can add some novel algorithmic modules, such as attention mechanisms and contrast learning, to improve the model effect based on the existing model in a targeted way. If we increase the sample size in the future, we can replicate the model framework consistent with this study and try to use more complex models to further optimize the model performance. In our study, we utilized non-thin MRIs commonly used in clinical practice, and we designed scenarios that reflect real reading situations to distinguish hippocampal sclerosis. This article presents the first step toward application, which is to validate the feasibility of our model and the materials used. For future applications and implementations, we aim to improve the accuracy and generalization ability of the model, as well as enhance its computing speed, reduce computing power and memory requirements, and develop application software. Additionally, we plan to consider automatic pushing to make it more accessible for primary hospitals.

There are some limitations of this study. First, the data were obtained only from the West China Hospital, which may limit the generalizability of the algorithm. Further external validation of our study is needed. The generalization ability of the model may need to be enhanced. However, this may not affect the feasibility of the main goal of our study, which is to use deep learning to discriminate HS based on MRI common sequences used in actual clinical diagnosis. Second, our study is currently only at the stage of validating feasibility. Therefore, the algorithm in its current form cannot be used directly in clinical practice yet. The algorithm needs to be further developed and validated in the context of the actual HS diagnosis process. Third, in the present study, we did not consider other health or medical-related data other than imaging data. And these data may indeed influence the model's judgment of hippocampal sclerosis (Mo et al., 2019). This study is an initial exploratory attempt to use deep learning methods for hippocampal sclerosis judgments on common non-thin MRI sequences used in real clinical practice. Based on the feasibility demonstrated in this study, our next study will add other health or medical-related data and fuse multimodal data of patients to synthesize the judgment of hippocampal sclerosis to further improve the accuracy and interpretability of the model. And we need to increase the study sample along with the clinical variables and increase the feature engineering part, because if there are too many variables and not enough model samples will lead to overfitting of the model. Fourth, our model only made the discrimination of hippocampal sclerosis or not, and did not give the severity of hippocampal sclerosis or the grade of sclerosis, so it could not provide more strong evidence to support the choice of treatment options. Therefore, another follow-up study of ours is to collect different severity levels of hippocampal sclerosis samples and build a multi-level classification model or severity scoring model. Fifth, our data are consistent with real clinical use and are not deliberately collected or finely designed. So our HS patients and HCs were collected over a large time span from 2009 to 2020, and scanner may indeed be upgraded, e.g., from 1.5T to 3.0T. However, the MRI materials included in our study were all 1.5-T scanners, differing only in the year of collection. And for deep learning, we would like to have multiple types of MRI as input to improve the generalization ability of the model. Besides, we observed that the ages of HS patients and HCs were not precisely matched. The age range of HCs was broader and encompassed the age range of HS patients, which better reflects the age distribution of a realistic normal population. However, this difference in age distribution may have introduced some bias in the model's discrimination ability. We will address this issue by continuing to collect more samples to reduce the age difference between the two populations and improve the model's stability. Finally, the differences reflected in our model results are numerical, not statistical.

## 5. Conclusion

Our HS-Net model has been developed to discriminate HS using non-thin MRI sequences commonly used by radiologists in real clinical diagnosis. The performance of the model (AUC = 0.89) confirms the feasibility of using deep learning and textures enhanced by the fractional differential method to discriminate HS from common clinical MRI sequences. This research supports the potential use of a deep learning-based tool for initial screening of HS in primary hospitals with limited MRI scanning capabilities, which may assist in guiding further diagnostic testing, medical visits, or referrals to specialized hospitals.

## Data availability statement

The data analyzed in this study is subject to the following licenses/restrictions: the data used to support the findings of this manuscript are restricted by the West China Hospital in order to protect patient privacy and avoid legal and ethical risks. Data are available from the West China Hospital for researchers who meet the criteria for access to confidential data. Requests to access these datasets should be directed to simaxiutian@wchscu.cn.

## Author contributions

JJ, JQ, and XS substantially contributed to conception and design. JQ and XS were responsible for acquisition of data. JJ contributed to analysis and interpretation of data, drafted the article, and revised it critically for important intellectual content. XS was responsible for the agreement to be accountable for all aspects of the work. All authors did critical revision of the manuscript for important intellectual content, contributed significantly to this work and have met the qualification of authorship. All authors contributed to the article and approved the submitted version.
