# Supplementary material for: Automated detection of hippocampal sclerosis using real-world clinical MRI images
Source: Front Neurosci. 2023 May 15;17:1180679. doi: 10.3389/fnins.2023.1180679 (PMC10225575; doi:10.3389/fnins.2023.1180679)
Supplement: Supplementary file 1 [file Data_Sheet_1.PDF]

Supplementary Material

1 SUPPLEMENTARY TABLES

Table S1. The comparison of the impact of varying v and c values in the FD method on experimental results.

	AUC	Accuracy	F1 Score	Precision	Recall
With different values of v					
$v = 2.0, c = 13$	0.710	66.44%	72.32%	65.31%	81.01%
$v = 2.2, c = 13$	**0.894**	**82.88%**	**84.08%**	**84.62%**	83.54%
$v = 2.4, c = 13$	0.723	71.92%	75.15%	72.09%	78.48%
With different values of c					
$v = 2.2, c = 3$	0.816	73.97%	77.91%	72.04%	84.81%
$v = 2.2, c = 8$	0.796	74.66%	77.84%	73.86%	82.28%
$v = 2.2, c = 13$	**0.894**	**82.88%**	**84.08%**	**84.62%**	83.54%
$v = 2.2, c = 18$	0.765	64.38%	72.63%	62.16%	**87.34%**

FD, fractional differential.
